# Effects of goat milk–based formula on development in weaned rats

**DOI:** 10.3402/fnr.v59.28610

**Published:** 2015-12-08

**Authors:** Meihong Xu, Liren Wei, Zhiyong Dai, Yanchun Zhang, Yong Li, Junbo Wang

**Affiliations:** 1 Department of Nutrition and Food Hygiene, School of Public Health, Peking University, Beijing, China; 2 Beijing Key Laboratory of Toxicological Research and Risk Assessment for Food Safety, Peking University, Beijing, China; 3 Ausntria Hyproca Dairy Group BV, Changsha, China

**Keywords:** infant formula, goat milk–based formula, development, immunity

## Abstract

**Objective:**

The aim of this article is to study the effect of goat milk–based formula (GMF) on development in weaned rats.

**Methods:**

One hundred Sprague-Dawley rats were randomly divided into five groups: control, 20% cow milk–based formula (CMF), and 5%-GMF, 10%-GMF, and 20%-GMF groups.

**Results:**

GMF did play an active role in accelerating body and femur length, but not body weight growth. Compared with the control, GMF had better cognitive, space, and locomotor activity. The level of IFN was increased in GMF groups, as well as the level of IL-2 and TNF was decreased in GMF groups.

**Conclusion:**

These results indicate that GMF has an effect on development and immunity improvability in weaned rats.

Goat milk is a food of high nutritional value, with high biological value protein, and a good source of short- and medium-chain fatty acids, minerals, and vitamins (1). The fat from this product has better digestibility, the protein has smaller allergenic potential, and this milk also has less lactose than cow milk (1). Furthermore, goat milk provides a better use of iron, which minimizes possible interactions between iron and other minerals such as calcium, phosphorus, and magnesium (2–6). More than a simple source of essential nutrients, goat milk contains many functional components, including lactoferrin, oligosaccharides, nucleotides, taurine, polyamines, and bioactive peptides (1). Developing new products that mimic human milk properties is a current target for infant formula manufactures. For the above benefit, goat milk–based formula (GMF) emerged as a new candidate.

When discussing GMF according to the existing literature, we found that they focused mainly on following points: First, the topics are usually on the comparison of nutrition content between goat, cow, and human milk (7–10). Second, most of the studies are clinical trials (11–13). The focus of animal studies was limited to the anti-inflammation, anti-allergy, anemia-healing, and the absorption of minerals, amino acids, etc. (2, 14–18). Third, the biological effect of GMF is not yet clear.

Therefore, the present article systematically discussed the effect of GMF on development in weaned rats. When rats were treated with GMF in the weaning period, immune effect should also be considered.

## Materials and methods

### Test substance

GMF and cow milk–based formula (CMF) were provided by Ausntria Hyproca Dairy Group BV (Hunan, China). The experimental diet was supplemented with ‘AIN93G’ to give 50 mg GMF, 100 mg GMF, 200 mg GMF, and 200 mg CMF of mixed MF g-1 diet. Dietary ingredients were thoroughly mixed in a mixer, made into pellets, and air-dried at room temperature. The rats Th1 (IL-2, TNF-α, and IFN-γ) bead-based multiplexed assay kits were purchased from Bender MedSystems Inc. (Burlingame, CA, USA).

### Experimental animal and housing conditions

In total, 100 male and female Sprague-Dawley rats (21 days old), weighing 40–60 g, were obtained from the Animal Service of Health Science Center, Peking University, Beijing. Rats were housed two per plastic cage with free access to chow and tap water in a specific pathogen-free filter-protected air-conditioned room with controlled temperature (21±23°C), relative air humidity (55±5%), and 12 h light/dark cycles (light on 07:30–19:30 h). All animals were handled in accordance with the guidelines of the National Institutes of Health (NIH Publication No. 85-23 revised 1985) and the guidelines of the Peking University Animal Research Committee (www.lab.pku.edu.cn).

### Experimental design

After a 1-week acclimation period, rats were randomly assigned to one of five groups (10 animals per sex per group): one control group, and four experimental groups. Control rats were fed with ‘AIN93G’ as rodent diet (Vital River Ltd. Co., Beijing). Rats in the four experimental groups were fed with 5% GMF, 10% GMF, 20% GMF, or 20% CMF (wt/wt) in the diet (Vital River Ltd. Co., Beijing). Animals were observed daily and weekly for adverse clinical signs. A detailed physical examination was conducted at least once during the pretreatment period, once weekly during test substance administration, and on the day of necropsy. Body weight, body length, and food consumption were recorded weekly. Diet utilization ratio was calculated using the following formula: Diet utilization ratio=[weight gain (g)/food consumption (g)]×100%.

On the 45th day, the urine and feces were collected in 24 h. The volume of urine and the weight of feces were recorded. Animals were fed with diets for 90 days. After 90 days, rats were anesthetized with CO_2_ inhalation and then sacrificed by cervical dislocation.

### Behavioral test

On day 45, spontaneous locomotor activity of rats was measured using the open field test (OFT). The apparatus, made of wood covered with impermeable formica, had a black floor of 100×100 cm^2^ and black walls of 30 cm high. The test room had a controlled illumination (dim light condition). The floor of the arena was divided into 16 equal squares. Each rat was placed in the center of the open field, and the latency time, the number of squares crossed with all paws (crossings), rearing, modification frequency, and modification were manually counted in a 3 min session. Locomotor activity was recorded between 13:00 and 17:00 h. The apparatus were cleaned with a solution of 10% ethanol between tests in order to hide animal clues.

### Hematology, serum biochemical evaluation

Blood samples were collected from femoral arteries for clinical pathology evaluations (hematology, serum chemistry) from rats immediately prior to the scheduled necropsy on day 90. All animals were fasted overnight prior to blood collection.

The following hematology parameters were analyzed using MLA Electra 1,400 CTM automated Coagulation Analyzer: white blood cells, lymphocyte count (LY#), monocytes count (MO#), neutrophilicgranulocyte count (GR#), mean corpuscular volume (MCV), mean corpuscular hemoglobin (MCH), hemoglobin (HGB), and eosinophil ratio (EO%).

The levels of alanine aminotransferase (ALT), aspartate aminotransferase (AST), total protein (TP), albumin (ALB), blood urea nitrogen (BUN), creatinine (CR), triglyceride (TG), globulins (GLB), ALB/GLB (A/G) and transferrin (TRF) in serum were detected by automatic biochemistry analyzer (Hitachi, Japan).

### Immune function assay

#### Immunoglobulin assay

An ELISA assay was used to measure serum immunoglobulin (IgA, T IgE, IgG, and IgM) and sIgA of intestinal mucosa immunoglobulin. Serum, containing Immunoglobulin A (IgA), T IgE, IgG, and IgM, is diluted with buffer containing polyethylene glycol (PEG) and mixed with specific polyclonal goat anti-IgA, anti-IgE, anti-IgG, or anti-IgM antiserum, forming a complex. This complex can be quantitated by measuring light absorbance at 700 nm. The sensitivity and the rate of forming the immune-complex can be increased by the addition of the polymer, PEG. sIgA followed the same method as serum immunoglobulin.

#### Bead-based multiplexed assay

A bead-based multiplexed assay (19, 20) was used to measure IL-2, IFN-γ, and TNF-α in serum. This assay used microspheres as the solid support for immunoassays and allowed the simultaneous quantification of several cytokines in a small sample volume. The test was performed and the analysis was carried out according to the manufacturer's instructions. Briefly, 25 µl serum samples, or the provided standards, were mixed with 25 µl antibody-coated capture beads and subsequently incubated with 50 µl biotin-conjugate second antibody mixture for 2 h at room temperature in a dark room. Beads were then added in wash buffer and centrifuged for 5 min at 400×*g*, and the supernatant discarded carefully, leaving 100 µl of liquid in each tube. This was repeated, and samples were further incubated with 50 µl streptavidin–phycoerythrin for 1 h at room temperature in a dark room. After two further centrifugation steps as mentioned above, the beads were re-suspended in 300 µl assay buffer and read on the BD FACSCalibur flow cytometer to determine cytokine concentration. The lower levels of detection for the 10 cytokines were between 0.7 and 15.7 pg/ml.

### Organ pathology

All animals were euthanized by carbon dioxide inhalation followed by exsanguination. Animals were necropsied and the external surface, all orifices, and the cranial, thoracic, abdominal, and pelvic cavities including viscera were examined. Organ weights were measured for the adrenal glands, brain, epididymides, heart, kidneys, liver, femurs, and ovaries with oviducts, spleen, testes, thymus and thyroids/parathyroids from all animals. Approximately 40 tissues were collected from each animal in the study for microscopy. The femur length and midpoint width were measured.

### Statistical analysis

Statistical analyses were performed using SPSS software (version 19.0, SPSS Inc., Chicago, IL, USA). Variances in the measurement data were checked for homogeneity by Bartlett's test. When the data were homogeneous, the one-way analysis of variance test and LSD methods were used. Tamhane's T2 test is used after data are transformed to analyze data among multiple groups if variances are unequal. All reported *P* values were two-sided. A value of *p*<0.05 was considered significant.

## Results

### General information

There was no remarkable difference in general condition and behavior between the control and treated rats of both sexes.

**Fig. 1:**
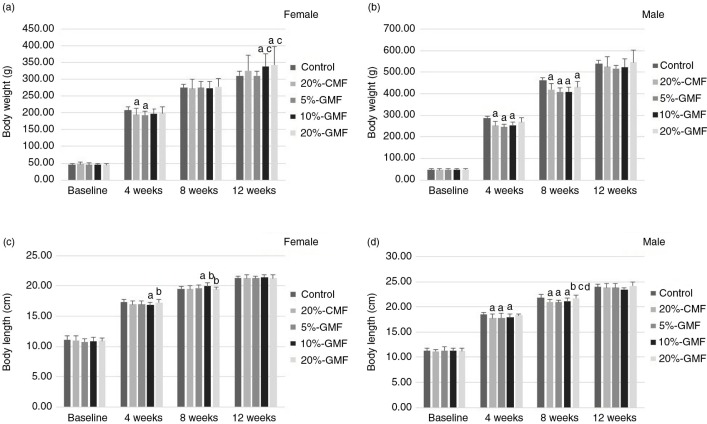
Effect of GMF on body weight and body length trends in Sprague-Dawley rats treated with different levels of GMF, shown by body weight (a, female; b, male) and body length (c, female; d, male). ^a^Statistical significance: *p*<0.05, compared with control; ^b^statistical significance: *p*<0.05, compared with 20%-CMF; ^c^statistical significance: *p*<0.05, compared with 5%-GMF; ^d^statistical significance: *p*<0.05, compared with 10%-GMF. GMF: goat milk–based formula.

As shown in Fig. 1a and b, at the age of 4 and 8 weeks the body weight of the GMF-treated male group was significantly lighter than the male control, with the exception of 20% GMF-treated in 4th weeks (*p*<0.05); meanwhile, the body weight of the GMF-treated female group was lighter than female control at the age of 4 weeks (*p*<0.05). However, at 12 weeks, the body weight of the 10%-GMF and 20%-GMF female groups was heavier than the control (*p*<0.05). In weeks 4 and 8, compared with the control, there were significant differences in body length in the 10%-GMF female group, as well as the CMF, 5%-GMF and 10%-GMF male groups (*p*<0.05, shown in Fig. 1c and d). However, at week 12, all the body weights and body lengths were at the same level in all GMF-treated, 20%-CMF, and control groups with the exception of 10% and 20% GMF-treated groups.

Compared with the control, only in week 4, the diet utilization ratio was increased with significant differences in all GMF and the 20%-CMF group for both female and male rats (*p*<0.05). The organs were weighed, and there were no significant differences in organ-to-body ratio among groups of either sex. The femur length in all GMF-treated female groups was longer than the 20%-CMF female and control female groups. There were no remarkable differences in midpoint width among the GMF-treated, 20%-CMF, and control groups (*p*>0.05).

### Locomotor activity indicators

The effect of GMF treatment on locomotor activity, by OFT, was shown in Table 1. There were significant differences in modification time between all GMF-treated female groups and the 20%-CMF female group. It was lower in the 20%-CMF female group (*p*<0.05). Oppositely, the modification time in the 20%-CMF male group was remarkably longer than that in the 10%-GMF male group (*p*<0.05). Similarly, the rearing frequency in the 20%-CMF female group was lower than that in the 20%-GMF female group; whereas it was higher than that in all GMF-treated male groups and the control.

**Table 1:** Locomotor activity in rats treated with different doses goat milk–based formula treatment by open field test

Sex	Group	*N*	Modification frequency	Latency time	Modification time	Rearing frequency	Number of crossing
Female							
	Control	10	1.19±1.40	6.67±2.96	9.24±4.69	15.76±8.21	2.14±2.29
	20%-CMF	10	1.60±1.67	8.05±3.49	4.70±2.41^a^	10.11±5.64	2.00±3.11
	5%-GMF	10	1.10±1.61	5.90±3.10	10.10±5.38^b^	10.52±8.59	0.71±1.55
	10%-GMF	10	1.90±1.38	7.19±6.87	10.29±6.03^b^	12.86±10.46	0.95±2.20
	20%-GMF	10	1.95±0.97	11.33±5.55	11.95±5.41^b^	17.14±11.60^b^^c^	1.90±2.90
Male							
	Control	10	1.25±0.97	7.83±2.86	6.00±2.92	4.75±2.63	0.58±1.17
	20%-CMF	10	2.25±2.01	8.33±3.89	7.67±2.74	11.75±5.68^a^	0.33±0.65
	5%-GMF	10	1.55±1.44	7.73±4.03	5.55±3.08	6.82±5.34^b^	1.18±2.04
	10%-GMF	10	2.92±2.28	6.50±3.09	5.00±3.05^b^	5.75±4.05^b^	0.33±0.89
	20%-GMF	10	1.58±1.38	6.33±2.06	6.92±3.12	7.75±3.57^b^	0.17±0.39

Data are mean±SD values.

a Statistical significance: *p*<0.05, compared with control;

b statistical significance: *p*<0.05, compared with 20%-CMF;

c statistical significance: *p*<0.05, compared with 5%-GMF.

### Hematology, serum biochemical indicators

Investigations of the hematology parameters were performed in Table 2. For the female part, in comparison with the control, the level of RBC# was increased in the GMF-treated and 20%-CMF groups. There was only one remarkable difference between 20%-GMF and control in RBC# (*p*<0.05). For the male part, there were many remarkable changes in MCV and MCH. Compared with the control, the level of MCV was significantly increased in all GMF-treated groups and the 20%-CMF group; moreover, the level of MCV in the 10%-GMF and 20%-GMF groups was lower than that in the 20%-CMF group (*p*<0.05). The level of MCH in the 5%-GMF and 20%-CMF groups was significantly higher than that in the control (*p*<0.05). There was remarkable difference between the 5%-GMF and 20%-CMF groups in GR#. In comparison with the control, the level of LY# was decreased in the GMF-treated groups; moreover, there were significant differences in the 10%-GMF and 20%-GMF groups. There was only one remarkable difference between the 20%-GMF male group with the 20%-CMF male group in MO# (*p*<0.05).

**Table 2:** Hematology parameters in rats treated with different doses of goat milk–based formula

			WBC	GR#	LY#	MO#	RBC#	MCV	HGB	MCH	EO%
Sex	Group	*N*	(10^9^/L)	(10^9^/L)	(10^9^/L)	(10^9^/L)	(10^12^/L)	(Fl)	(g/L)	(pg)	(10^9^/L)
Female											
	Control	10	5.77±1.73	1.74±0.53	3.87±1.45	0.01±0.03	7.28±0.67	55.22±1.97	156.11±12.40	21.46±0.60	0.14±0.05
	20%-CMF	10	6.58±1.73	2.26±1.27	4.01±1.06	0.11±0.32	7.41±0.37	53.94±2.43	155.38±4.69	21.01±1.04	0.19±0.06
	5%-GMF	10	6.10±1.71	1.98±1.00	3.93±1.37	0.00±0.00	7.50±0.43	55.19±2.24	162.50±6.30	21.71±0.72	0.20±0.11
	10%-GMF	10	5.41±2.12	1.63±0.65	3.58±1.57	0.03±0.05	7.51±0.45	54.94±2.50	161.88±8.06	21.60±0.71	0.19±0.08
	20%-GMF	10	6.17±2.08	2.01±1.05	3.93±1.05	0.01±0.04	7.75±0.28^a^	53.66±1.30	162.29±6.50	20.93±0.77	0.21±0.15
Male											
	Control	10	9.14±3.01	3.49±1.61	5.45±1.82	0.05±0.08	8.30±1.08	50.45±1.59	158.25±20.37	19.10±0.81	0.15±0.08
	20%-CMF	10	9.70±1.69	4.38±1.61	5.00±1.54	0.15±0.19	7.91±0.46	54.64±2.19^a^	162.88±9.06	20.59±0.55^a^	0.18±0.09
	5%-GMF	10	7.70±2.21	2.85±0.98^b^	4.59±1.50	0.09±0.06	7.88±0.78	52.95±2.15^a^	158.50±11.31	20.18±0.96^a^	0.18±0.10
	10%-GMF	10	9.28±2.52	3.66±1.32	3.36±2.08^a^	0.08±0.21	8.39±0.32	52.71±2.01^a^^b^	160.62±6.91	19.16±0.49^b^^c^	2.13±2.24
	20%-GMF	10	8.93±3.09	2.91±1.82	3.51±2.28^a^	0.03±0.07^b^	8.22±1.01	52.36±1.57^a^^b^	158.88±19.93	19.35±1.05^b^^c^	2.23±2.72

Data are mean±SD values.

a Statistical significance: *p*<0.05, compared with control;

b statistical significance: *p*<0.05, compared with 20%-CMF;

c statistical significance: *p*<0.05, compared with 5%-GMF. WBC: white blood cell; GR#: neutrophilicgranulocyte count; LY#: lymphocyte count; MO#: monocytes count; RBC#: red blood cell count; MCV: mean corpuscular volume; HGB: hemoglobin; MCH: mean corpuscular hemoglobin; EO%: eosinophil ratio.

Comparison of mean serum chemistry values from the GMF treated groups with the values from normal control is shown in Table 3. The significant differences of ALT, compared with control, were observed in all GMF-treated groups and the 20%-CMF group, in either sex group, with the exception of 20% GMF-treated male group; meanwhile, significant differences of AST were observed in the 20%-GMF and 20%-CMF groups, both male and female groups (*p*<0.05). The significant differences of ALB were observed in the 5%-GMF and 10%-GMF female groups, compared with the control and 20%-CMF.

**Table 3:** Serum biochemistry parameters in rats treated with different doses of goat milk–based formula

			TP	ALB	GLB	AST	ALT	TRF	BUN	Cr	TG
Sex	Group	*N*	(g/L)	(g/L)	(g/L)	(U/L)	(U/L)	(mg/dl)	(mmol/L)	(µmmol/L)	(mmol/L)
Female											
	Control	10	74.56±2.68	37.96±1.48	36.60±1.72	161.50±31.74	35.00±6.07	165.09±13.05	5.96±0.70	31.35±3.24	0.85±0.18
	20%-CMF	10	76.04±4.52	37.61±2.99	38.43±1.92	97.63±15.63^a^	19.00±3.34^a^	180.51±18.64	7.00±2.88	36.43±22.55	1.00±0.34
	5%-GMF	10	81.68±7.65	41.55±3.23^a^^b^	40.13±4.63	149.00±36.93^b^	21.25±3.01^a^	166.99±19.33	7.41±2.15	29.25±9.89	1.36±0.67
	10%-GMF	10	82.04±5.87	40.80±2.63^a^^b^	41.24±3.31	182.57±36.61^b^^c^	27.43±3.78^a^^b^^c^	177.30±19.57	6.29±1.60	28.42±3.10	1.76±0.88
	20%-GMF	10	77.90±5.02	39.54±2.37	38.36±2.96	98.63±20.21^a^^c^^d^	20.25±2.49^a^^d^	175.88±13.98	6.07±1.65	31.37±5.43	1.03±0.44
Male											
	Control	10	73.14±3.54	35.86±1.10	37.28±2.89	193.88±26.47	47.38±8.53	205.66±34.31	6.09±0.63	21.47±2.96	1.37±0.48
	20%-CMF	10	73.61±4.09	36.45±1.59	37.16±2.99	140.62±22.10^a^	29.25±5.12^a^	204.16±42.43	6.93±1.60	20.46±4.56	1.44±0.70
	5%-GMF	10	76.24±2.96	36.85±1.08	39.39±2.34	207.88±28.17^b^	38.63±7.67^a^^b^	183.96±24.85	7.38±2.26	22.36±4.28	1.14±0.39
	10%-GMF	10	74.05±4.43	36.53±1.71	37.53±2.94	201.62±40.71^b^	36.00±4.57^a^	189.09±29.70	6.66±1.22	24.19±8.58	1.27±0.44
	20%-GMF	10	75.78±3.66	36.65±1.84	39.13±1.98	146.12±41.95^a^^c^^d^	40.13±7.08^b^	189.75±25.29	6.74±1.68	19.05±4.62	1.21±0.43

Data are mean±SD values.

a Statistical significance: *p*<0.05, compared with control;

b statistical significance: *p*<0.05, compared with 20%-CMF;

c statistical significance: *p*<0.05, compared with 5%-GMF;

d statistical significance: *p*<0.05, compared with 10%-GMF. ALT: alanine aminotransferase; AST: aspartate aminotransferase; TP: total protein; ALB: albumin; UA: serum uric acid; BUN: blood urea nitrogen; CR: creatinine; TC: total cholesterol; TG: triglyceride; HDL-C: high-density lipoprotein-cholesterol; GLU: glucose.

### Immune parameters

As shown in Table 4 female part, in comparison with the control, the level of TNF were decreased with significant differences in all GMF groups and the 20%-CMF group (*p*<0.05). Similarly, the level of IL-2 were remarkable decreased in 20%-CMF and 10%-GMF male group. The level of IFN was remarkable increased in the 20%-CMF and 10%-GMF groups, compared with the control (*p*<0.05). Compared with the 20%-CMF group, all the cytokine levels were at the same level in the GMF group. However, for the male part, there were no significant differences between the control and all GMF groups.

**Table 4:** Immunity parameters in rats treated with different doses of goat milk–based formula

			IFN	IL-2	TNF	SIgA	IgA	IgG	IgM
Sex	Group	*N*	(µg/ml)	(µg/L)	(ng/ml)	(mg/dl)	(mg/dl)	(mg/dl)	(mg/dl)
Female									
	Control	10	59.51±28.09	46.87±14.30	35.23±7.72	0.31±0.14	0.45±0.18	62.37±15.00	18.56±3.74
	20%-CMF	10	99.13±10.79^a^	30.56±4.86^a^	22.64±3.13^a^	0.38±0.30	0.56±0.12	54.17±12.14	26.86±19.36
	5%-GMF	10	81.75±11.32	36.74±6.06	26.58±4.31^a^	0.29±0.14	0.52±0.15	55.74±4.85	26.84±8.71
	10%-GMF	10	92.17±23.17^a^	33.87±10.04^a^	23.07±7.53^a^	0.21±0.12	0.55±0.11	59.53±9.35	25.61±6.04
	20%-GMF	10	82.79±6.11	38.15±3.32	24.36±2.22^a^	0.42±0.07	0.43±0.18	56.65±8.10	25.98±10.34
Male									
	Control	10	67.11±13.29	42.73±7.39	30.19±5.10	0.52±0.11	0.79±0.17	56.62±14.49	21.64±5.86
	20%-CMF	10	66.52±24.07	44.48±10.94	29.92±4.76	0.46±0.14	0.77±0.17	52.02±9.77	24.43±6.09
	5%-GMF	10	47.20±27.04	53.84±12.53	57.39±43.47	0.61±0.33	0.64±0.20	53.64±7.82	20.52±7.86
	10%-GMF	10	61.59±10.01	46.30±4.05	32.83±2.97	0.31±0.11	0.81±0.11	52.26±5.22	19.99±6.64
	20%-GMF	10	78.32±25.74	39.12±11.71	27.85±7.67	0.26±0.14	0.69±0.21	58.97±4.13	25.78±5.74

Data are mean±SD values.

a Statistical significance: *p*<0.05, compared with control.

The effect of GMF on immunoglobulin in rats was obtained by testing IgA, T IgE, IgG, and IgM in serum, and sIgA in intestinal mucosa. Although, compared with the 20%-CMF group, the level of IgG in all GMF groups and the level of sIgA in the 20%-GMF group are higher, there were no significant differences (*p*>0.05). In general, there was no remarkable change of GMF in immunoglobulin in either sex (Table 4).

## Discussion

This study, on the developmental effect of GMF in the weaned period, aims at augmenting the currently available data. It indicated that GMF had the effect on development and immunity improvability. To the best of our knowledge, this is the first GMF study on development in weaned rats.

As a whole, it can be seen in this study that GMF did not play an active role in accelerating body weight growth, but did for body and femur length. At the first 8 weeks, the body weight of the GMF-treated male group was significantly lighter than the control. At week 12, all the body weights and body lengths were at the same level in all GMF-treated, 20%-CMF, and control groups, both female and male. This is consistent with the result of C Grant's study (12). However, at weeks 4 and 8, compared with the 20%-CMF group, there were significant differences in body length in 10% and 20% GMF female groups, as well as the 20% GMF male group. The rapid growth period of body weight is in the first 5 weeks, as in early childhood. It indicated that GMF is effective on growth in the infant stage, rather than in later periods. The femur length in all GMF-treated female groups was longer than the 20%-CMF female and control female groups. It presents a good dose–response relationship. There is a close relationship between body length and the growth of bones. Goat milk is the source of natural small molecule calcium, with a high quality. Moreover, casein, one of the main proteins found in goat milk, contains the bioactive protein casein phosphopeptides (CPPs). It has been suggested that CPPs increase calcium solubility by binding to calcium in the small intestine, where passive calcium absorption takes place (21–23). Fat of goat milk may also contribute to increased calcium absorption (24). All of these things are good for increasing the bone density, and then bone strengthening. Compared with the females, there is an inferior effect in male rats. This may be related to the development character and hormone level of female rats (25). Furthermore, no clinical adverse effect changes were revealed on organ weight and function of GMF-treated groups.

We analyzed the whole hematology parameters. Compared with the control, there was remarkable change in the level of RBC#, MCV, and MCH, which indicated GMF may have an anti-anemia ability (26). Additionally, compared with the control, there were no remarkable changes of serum biochemical data in the GMF-treated groups with the exception of a statistically significant decrease in AST and ALT levels. It is indicated GMF may have the effect of protecting the liver, which may be in part due to its high content of nucleotides.

OFT is now one of the most popular procedures in animal psychology. In such a situation, rodents spontaneously prefer the periphery of the apparatus to activity in the central parts of the open field. Indeed, rats walk close to the walls, a behavior called thigmotaxis. Normally, rats’ instinct will prompt them to avoid empty fields (27). In the present paper, we use OFT to measure the spontaneous locomotor activity of rats. It indicated the rats, treated by GMF, have a better spatial cognitive ability, compared to CMF and the control, especially in females. However, this needs further research such as step-down tests and Morris water maze.

In this study, the level of IFN was increased in GMF groups, as well as the level of IL-2 and TNF was decreased in GMF groups. GMF may have the effect of enhancement on immunity function. However, EO#, allergy-reflection index, did not have a statistical difference. For this part, further studies need to be performed using allergy models.

## Conclusions

GMF may play roles in the promotion of early growth, development, and immunity after weaning, especially in female rats. Further research is needed to use specific models to elucidate the effect of GMF on neurobehavior or allergy.
